# Comparative Transcriptome Analysis of Latex Reveals Molecular Mechanisms Underlying Increased Rubber Yield in *Hevea brasiliensis* Self-Rooting Juvenile Clones

**DOI:** 10.3389/fpls.2016.01204

**Published:** 2016-08-09

**Authors:** Hui-Liang Li, Dong Guo, Jia-Hong Zhu, Ying Wang, Xiong-Ting Chen, Shi-Qing Peng

**Affiliations:** Key Laboratory of Biology and Genetic Resources of Tropical Crops, Ministry of Agriculture, Institute of Tropical Bioscience and Biotechnology, Chinese Academy of Tropical Agricultural Sciences, HaikouChina

**Keywords:** *Hevea brasiliensis*, self-rooting juvenile clone, latex, transcriptome, epigenetic

## Abstract

Rubber tree (*Hevea brasiliensis*) self-rooting juvenile clones (JCs) are promising planting materials for rubber production. In a comparative trial between self-rooting JCs and donor clones (DCs), self-rooting JCs exhibited better performance in rubber yield. To study the molecular mechanism associated with higher rubber yield in self-rooting JCs, we sequenced and comparatively analyzed the latex of rubber tree self-rooting JCs and DCs at the transcriptome level. Total raw reads of 34,632,012 and 35,913,020 bp were obtained from the library of self-rooting JCs and DCs, respectively, by using Illumina HiSeq 2000 sequencing technology. *De novo* assemblies yielded 54689 unigenes from the library of self-rooting JCs and DCs. Among 54689 genes, 1716 genes were identified as differentially expressed between self-rooting JCs and DCs via comparative transcript profiling. Functional analysis showed that the genes related to the mass of categories were differentially enriched between the two clones. Several genes involved in carbohydrate metabolism, hormone metabolism and reactive oxygen species scavenging were up-regulated in self-rooting JCs, suggesting that the self-rooting JCs provide sufficient molecular basis for the increased rubber yielding, especially in the aspects of improved latex metabolisms and latex flow. Some genes encoding epigenetic modification enzymes were also differentially expressed between self-rooting JCs and DCs. Epigenetic modifications may lead to gene differential expression between self-rooting JCs and DCs. These data will provide new cues to understand the molecular mechanism underlying the improved rubber yield of *H. brasiliensis* self-rooting clones.

## Introduction

The rubber tree (*Hevea brasiliensis* Muell. Arg.) is commercially cultivated to produce natural rubber (*cis*-1,4-polyisoprene). As cutting propagation from a selected mother tree is impossible, the rubber tree is presently exploited for rubber production and is propagated by grafting axillary buds (scions) of elite clones onto unselected seedlings (rootstocks) (Clément-Demange et al., 2007; Hua et al., 2010). However, the rubber tree is a cross-pollinated plant; rootstock plants obtained by germinating open pollinated seeds harvested from the fields are highly heterozygous, which can lead to stock–scion interactions. Large intraclonal variations observed in the growth and yield of bud-grafted clones of the rubber tree are attributed to the genetic heterogeneity of the root stocks (Chandrashekar et al., 1997; Clément-Demange et al., 2007; Hua et al., 2010). Novel plantlets derived from somatic embryos of anthers and internal integuments of immature fruits of *H. brasiliensis* were obtained through primary somatic embryogenesis (SE)) in the early 1980s (Wang et al., 1980; Carron and Enjalric, 1982). The plantlets derived from somatic embryos of anthers were designated as self-rooting juvenile clones (JCs) (Chen et al., 2002; Clément-Demange et al., 2007; Hua et al., 2010). Self-rooting JCs demonstrated better performance in rubber yield, higher laticifer number, and larger trunk girth than those of donor clones (DCs) (Liu et al., 1985; Yang and Mo, 1994; Chen et al., 1998, 2002; Yuan et al., 1998). Therefore, self-rooting JCs are promising planting materials for rubber production (Chen et al., 2002; Carron et al., 2007; Hua et al., 2010).

Since the 1990s, a great deal of effort has been invested to understand the difference between self-rooting JCs and DCs. Initially, self-rooting JCs are thought to be superior over corresponding DCs in latex flow and latex regeneration (Yang and Mo, 1994; Yuan et al., 1998), in which their average stem girth at 50 cm above ground is 109 to 135% that of DCs (Chen et al., 1998, 2002). Up to 176 differentially expressed genes (DEGs) in the latex between self-rooting JCs and DCs have been characterized by suppression subtractive hybridization method (Li et al., 2011b, 2014). Systematic analyses of the DEGs between self-rooting JCs and DCs suggest that rubber biosynthesis, production, and scavenging of reactive oxygen species (ROS) may provide significant functions in high-yielding self-rooting JCs. Twenty-four differentially expressed proteins in the latex between self-rooting JCs and DCs have been isolated by 2 D polyacrylamide gel electrophoresis and mass spectrometry methods. These proteins were classified as stress/defense response, rubber biosynthesis, metabolism and energy, protein metabolism, signal transduction transcription, and post-transcription (Li et al., 2011a). To date, many gaps exist in the knowledge and understanding of the molecular mechanism underlying the difference between self-rooting JCs and DCs.

The natural rubber is synthesized and stored in laticifer, a specific tissue densely located in the secondary phloem of rubber tree trunk (d’Auzac et al., 1995; Puskas et al., 2006). The latex is the cytoplasm of laticifer cells and is used to refine natural rubber (d’Auzac et al., 1995). To study the molecular mechanism associated with higher rubber yield in self-rooting JCs, we selected latex for mRNA isolation. A comparative analysis of latex transcriptome between self-rooting JCs and DCs was performed. In the present study, we reported the global changes in gene expression in the latex between self-rooting JCs and DCs, thus uncovering the molecular mechanism underlying the higher rubber productivity in self-rooting JCs.

## Materials and Methods

### Plant Material

Ten-year-old trees of rubber tree cultivar CATAS7-33-97 self-rooting JCs and DCs, as well as HAIKEN 2 self-rooting JCs and DCs were planted at the Experimental Farm of the Chinese Academy of Tropical Agriculture Science and opened for tapping on the s/2 d/3 system (the length of tapping line was half of the stem girth, and the tapping frequency was 2-day intervals) in 2012. Up to 10 mL of latex from each tested tree were collected. The latex from 10 trees of self-rooting JCs or DCs was mixed well, and then the samples were immediately stored at -80°C until RNA extraction.

### RNA Extraction and Sequencing

Total RNA was extracted as previously described (Tang et al., 2007). Total RNA of CATAS7-33-97 self-rooting JCs and DCs was purified using RNeasy mini spin columns (Qiagen, Valencia, CA, USA). Paired-end Illumina cDNA libraries were generated following the manufacturer’s instructions for mRNASeq sample preparation (Illumina Inc., San Diego, CA, USA). The library quality was assessed using the 2100 Bioanalyzer (Agilent Technologies, Palo Alto, CA, USA). The libraries were deep sequenced using Illumina HiSeqTM 2000 (Sano Geno Max Co., Ltd., Beijing, China). The original image datasets were transferred into the sequence datasets by base calling. Clean reads were obtained by removing the adaptor sequence, empty tags, tags with only one copy, and low-quality sequences.

### Transcriptome *De novo* Assembly, Annotation, and Classification

Transcriptome *de novo* assembly was performed using Trinity^1^ (release 20140413) (Grabherr et al., 2011). Adjacent contigs were constructed into scaffolds by read mate pairs. Within the scaffold, the connected contigs used ‘N’ to represent unknown sequences and insert size information. Finally, paired-end information was used to fill the gap of scaffolds and to obtain the extended sequences with fewer Ns, which were defined as unigenes for further analysis. All unigenes were used for BLAST searches (*E* < 1E-5) against databases in NCBI Nr^2^ and kyoto encyclopedia of genes and genome (KEGG)^3^. The optimal aligning results were selected for unigene annotation. The aligning results were selected with an order of Nr, KEGG. To classify the unigenes, the Blast2GO program was used to obtain GO annotation based on molecular function, biological process, and cellular components (Conesa et al., 2005). All unigenes were also aligned to the COG database (Ye et al., 2006) to predict possible functions and the KEGG pathway database (Kanehisa et al., 2008) to perform pathway assignments.

### Analysis of Unigene Expression Difference

Reads per kb per million reads (RPKM) method was applied to calculate the expression of the unigenes (Mortazavi et al., 2008). A complex algorithm was developed to identify DEGs between self-rooting JCs and DCs. Raw clean reads in each library were normalized to RPKM to obtain the normalized gene expression level. In our analysis, we selected DEGs with FDR of ≤0.001 and ratio of >2 to conduct GO functional analysis and KEGG pathway analysis. “Upregulated” means the level of gene transcripts were higher in self-rooting JCs, whereas “downregulated” means the level of gene transcripts were higher in DCs.

### Validation of Gene Expression by Quantitative PCR (qPCR)

Fifteen genes were selected for validation by Quantitative PCR (qPCR). qPCR was performed in a Mx3005P Real-Time PCR System by using a SYBR Premix kit (Takara, Dalian, China). Fifteen specific primer pairs were designed (Supplementary Table S1). *HbACT7* was used as an internal control. qPCR conditions were described as follows: 30 s at 95°C for denaturation, 40 cycles for 10 s at 94°C, 30 s at 60°C, and 15 s at 72°C for amplification. Data obtained from qRT-PCR were clustered in accordance with the instructions provided by Stratagene (Santa Clara, CA, USA). All relative expression data were based on three individual reactions. Analysis of variance (ANOVA) was used to compare the statistical difference based on Fisher’s LSD test. Means were considered significantly different based on the *P* value (*P* < 0.05 and *P* < 0.01), which are indicated by asterisks (*) when *P* < 0.05, or indicated by (**) when *P* < 0.01.

## Results

### Sequencing and *De novo* Assembly of Latex Transcriptome

Transcriptome sequencing through RNA-Seq is a relatively straightforward approach that allows exploration of gene expression profiles at a global scale. To provide a comprehensive overview of the molecular mechanism associated with higher rubber yield in self-rooting JCs at a transcriptional level, we sequenced two latex cDNA libraries constructed from CATAS7-33-97 self-rooting JCs and DCs by using the Illumina HiSeq 2000 platform. Total raw reads of 33,502,206 and 33,487,380 bp were generated from the library of JCs and DCs, respectively. After filtering and removing the reads containing adapter sequences and unknown nucleotides, as well as low-quality reads, total clean reads of 30,622,397 and 30,705,857 bp were obtained with 4.00 G and 4.01 G nucleotides, correspondingly. Afterward, these clean reads were *de novo* assembled in 54689 unigenes with a mean length of 1057 bp and N50 length of 1808 bp (**Table 1**). All clean reads will be deposited in the NCBI Sequence Read Archive (SRA) (data have been submitted to NCBI and are being processed, will be under the accession number of SRR3741688).

**Table 1:** Summary for *Hevea brasiliensis* transcriptome.

Number of Unigene	54689
Large unigene (≥1000 bp)	20913
Max unigene length (bp)	16589
Mean unigene length (bp)	1057
N50 length (bp)	1808
GC (%)	41.20%
Total base (MB)	55.18

### GO Classification

**FIGURE 1:**
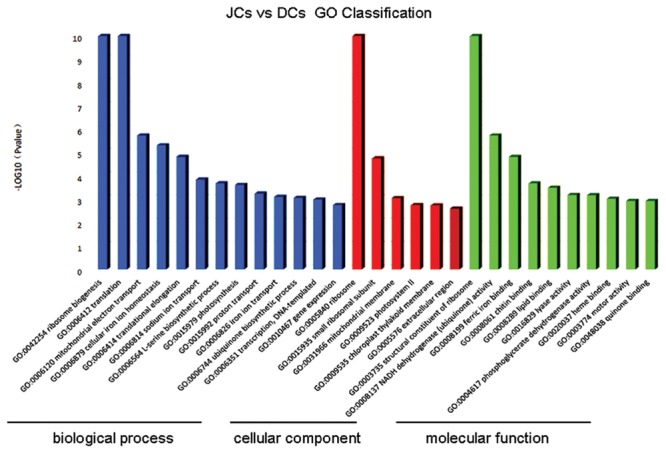
**Ontology classification of differential expression genes in self-rooting JCs and DCs latex of rubber tree.** Distribution of expressed genes in self-rooting JCs and DCs with the categories of biological process, cellular component, and molecular function.

All the unigenes were used to match against both the NCBI Non-redundant (Nr) by using BLASTX program with an E-value threshold of 1E-5, of which 33,602 (61.4%) unigenes were positively matched with the Nr protein database. Comparative analysis of transcriptome data of self-rooting JCs versus DCs under the conditions of FDR ≤0.001 and | log_2_ Ratio|≥1 showed 1,716 unigenes with differential expression, of which 1407 unigenes were upregulated, whereas 309 unigenes were downregulated (Additional File 1: Supplementary Table S2). “Upregulated” means the level of gene transcripts was higher in self-rooting JCs, whereas “downregulated” means the level of gene transcripts was higher in DCs. Among the 1,716 unigenes with differential expression, 906 (52.8%) showed significant homology to genes with known or partially known functions. The 906 DEGs with known or partially known functions were assigned to three main categories: biological process, cellular component, and molecular function (**Figure 1**). A total of 29 GO subcategories were represented under the three major categories. The majority of the GO annotations were in the biological process category, assigned to 23.7% distinct gene sequences, followed by the cellular component category for 21.1% distinct gene sequences, and the molecular function category for 55.2% distinct gene sequences. The major subcategories are shown in **Figure 1**. The five major biological process subcategories were ribosome biogenesis (GO:0042254), translation (GO:0006412), mitochondrial electron transport (GO:0006120), cellular iron ion homeostasis (GO:0006879), and translational elongation (GO:0006414); the two major cellular component subcategories were “ribosome” (GO:0005840) and “small ribosomal subunit” (GO:0015935); and the three major molecular function subcategories were “phosphoglycerate dehydrogenase activity” (GO:0004617G), heme binding (GO:0020037), and “motor activity” (GO:0003774). However, only 79.72% of DEG sequences were assigned with these GO terms, possibly because of the large number of uninformative gene descriptions of these plant protein hits.

### Kyoto Encyclopedia of Genes and Genomes Pathway Mapping

**FIGURE 2:**
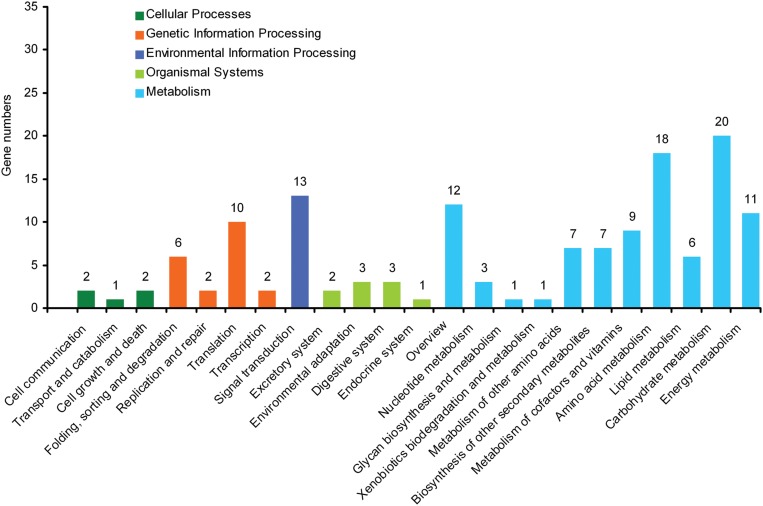
**Kyoto encyclopedia of genes and genomes (KEGG) orthology of differential expression genes between self-rooting JCs and DCs**.

By mapping enzyme numbers to the reference canonical pathways, DEG sequences were assigned to 23 KEGG pathways (**Figure 2**). The pathways most represented by unique sequences were cellular processes (5 members), genetic information processing (20), environmental information processing (13), organismal systems (9), and metabolism (95). Overall, these annotations provide a valuable resource to investigate the specific processes, structures, functions, and pathways involved in the difference between self-rooting JCs and DCs.

### Functional Analysis of the Enriched Categories in Self-Rooting JCs and DCs

**FIGURE 3:**
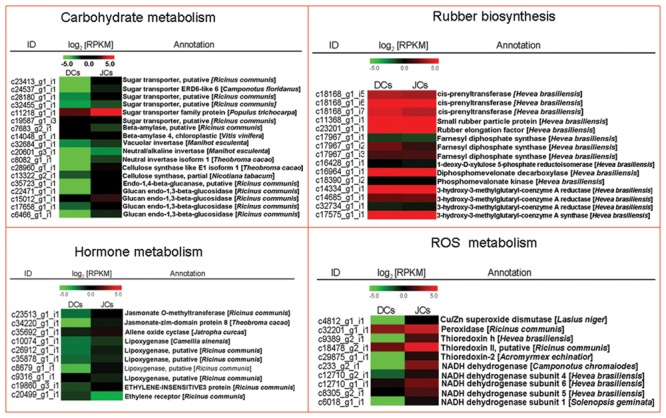
**Heat map of genes involved in carbohydrate metabolism, rubber biosynthesis, hormone metabolism, and ROS metabolism**. The values in red and green indicate log2 fold increases and decreases, respectively.

Kyoto encyclopedia of genes and genome analysis of all the differentially expressed unigenes showed that the mass of categories was differentially enriched between self-rooting JCs and DCs. Carbohydrate metabolism can provide a carbon skeleton to form various organic compounds (Chao et al., 2015). DEG analysis showed that plenty of unigenes associated with carbohydrate metabolism (e.g., unigenes encoding sugar transporter and starch degradation) were significantly different between self-rooting JCs and DCs. Six differentially expressed unigenes related to sugar transporter were notably upregulated in self-rooting JCs. Two unigenes encoding for beta-amylase and three unigenes for invertase were detected in DEG data and upregulated in self-rooting JCs. Two unigenes encoding for cellulose synthase and four unigenes for endo-1,4-beta-glucanase were upregulated in DEG data (**Figure 3A**). Jasmonates were pivotal to the secondary laticifer differentiation, whereas ethylene was the most effective in prolonging the duration of latex flow upon tapping in rubber tree (Hao and Wu, 2000; Zhu and Zhang, 2009). In the present study, the upregulated unigenes in the JA signaling pathway in self-rooting JCs included five unigenes for lipoxygenase, one for allene-oxide cyclase, one unigene for jasmonate O-methyltransferase, and one unigene for jasmonate ZIM-domain. In the ethylene signaling pathway, one unigene for ethylene-insensitive 3 was upregulated, and one unigene for ethylene receptor was also upregulated in self-rooting JCs (**Figure 3**). In addition, the duration of latex flow is reported to be dependent on the integrity of lutoids. Antioxidants antagonized ROS-caused membrane lipid peroxidation and played a role in keeping the integrity of lutoid (Chrestin et al., 1997). The unigenes encoding superoxide dismutase (SOD), namely, thioredoxin, peroxidase, and NADH dehydrogenase, were upregulated in self-rooting JCs (**Figure 3**). By contrast, most unigenes related to categories of rubber biosynthesis were not significantly different between self-rooting JCs and DCs (**Figure 3**).

### DEGs of Transcription Factors

**FIGURE 4:**
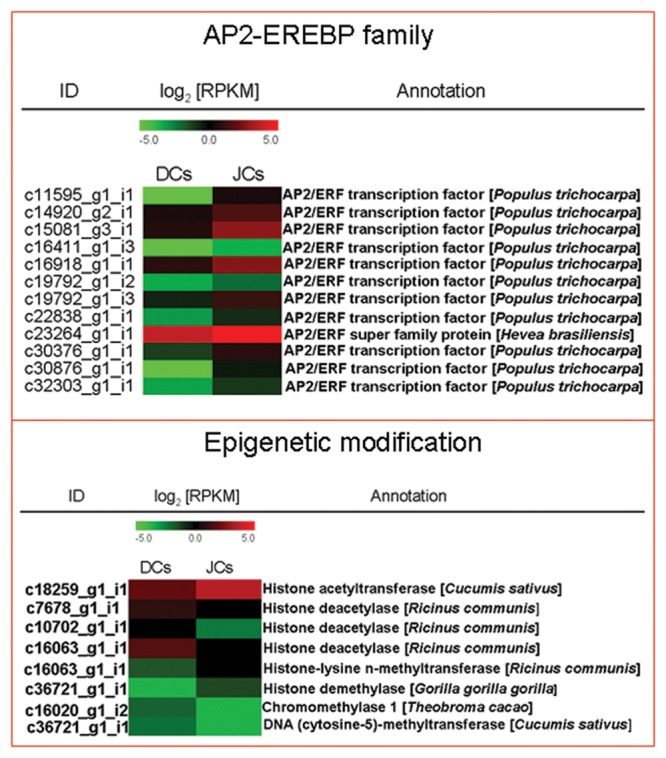
**Heat map of genes involved in AP2-EREBP familial and epigenetic modifications.** The values in red and green indicate log2 fold increases and decreases, respectively.

Several transcription factors (TFs) encoding genes were also differentially expressed between self-rooting JCs and DCs. These genes belong to more than eight different TF families, including C2H2 (5 genes), AP2-EREBP (12 genes), NAC (20 genes), MYB (10 genes), homeobox (15 genes), WRKY (17 genes), bZIP (1 gene), and MADS-box (7 genes). The majority of stress-responsive genes involved in ethylene responses have been classified in the TF family, AP2-EREBP. In our study, we noted that 12 genes related to the AP2-EREBP family are more highly expressed in self-rooting JCs than in DCs (**Figure 4**).

### Expression Patterns of Genes Related to Epigenetic Modifications

Self-rooting JCs derived from somatic embryos of anthers of *H. brasiliensis* was obtained through primary SE. *In vitro* plant regeneration can cause genetic and epigenetic variations (Machczyńska et al., 2015). Eight genes encoding epigenetic modification enzymes were also differentially expressed between self-rooting JCs and DCs (**Figure 4**), including DNA (cytosine-5)-methyltransferase (1 gene), chromomethylase (1 gene), histone acetyltransferase (1 gene), histone deacetylase (3 genes), histone-lysine *N*-methyltransferase (1 gene), and histone demethylase (1 gene).

### Confirmation of Gene Expression

**FIGURE 5:**
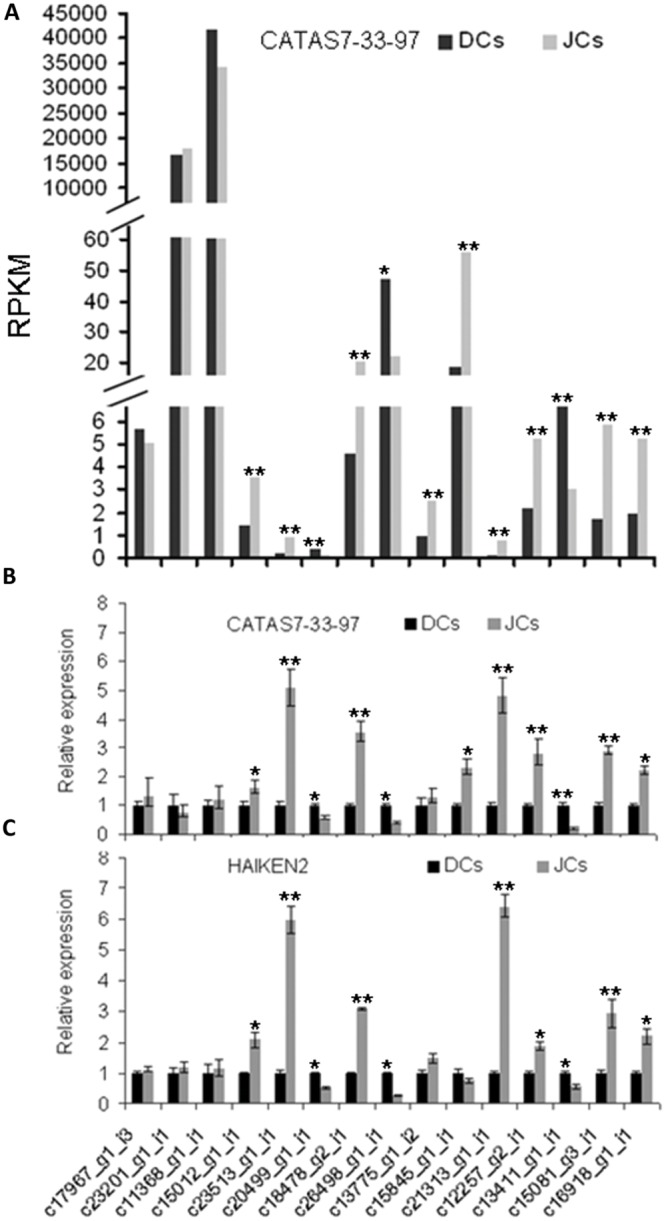
**Quantitative PCR (qPCR) confirmation of DEGs between self-rooting JCs and DCs. (A)** DEGs of differential expression genes between CATAS 7–33–97 self-rooting JCs and its DCs. **(B)** qPCR analysis of differential expression genes between CATAS 7–33–97 self-rooting JCs and its DCs. **(C)** qPCR analysis of differential expression genes between HAIKEN2 self-rooting JCs and its DCs. Asterisks indicate significant differences (Student’s *t*-test: *p < 0.05; **p < 0.01).

To validate the transcriptome results, 15 genes (11 of the 15 unigenes showed differential expression between CATAS 7–33–97 self-rooting JCs and DCs) were verified by qPCR. These 15 genes were involved in natural rubber biosynthesis, jasmonate synthesis, and transcription. Their expression patterns were fitted well with that obtained by transcriptome analysis (**Figures 5A,B**).

To further test the difference in gene expression between self-rooting JCs and their DCs, we analyzed the expression of the 15 genes in another rubber tree cultivar’s (HAIKEN 2) self-rooting JCs and DCs by using qRT-PCR. As shown in **Figure 5C**, their expression patterns were similar to those obtained from CATAS 7–33–97 self-rooting JCs and DCs.

## Discussion

The latex parameters, i.e., dry rubber content (DRC), sucrose, thiols and inorganic phosphorus contents, revealed direct correlations with the production in rubber trees. Some physiological parameters of the latex between self-rooting JCs and DCs had been compared. Self-rooting JCs showed similar thiol contents to DCs, whereas the sucrose and inorganic phosphorus contents and DRC were significantly higher than DCs. Plugging index and lutoid bursting index of self-rooting JCs were significantly lower than DCs (Yuan et al., 1998). These data suggest that self-rooting JCs are superior to DCs in both latex regeneration and latex flow. Although the analysis of latex physiological parameters provided clues about the mechanisms of higher rubber yield in self-rooting JCs, the differences are unclear in the molecular events occurring in the laticifers between self-rooting JCs and DCs.

In this study, we have first profiled the gene expression between self-rooting JCs and DCs by using Illumina sequencing technology. This transcriptome dataset will serve as an important public information platform for the discovery of the mechanisms underlying the higher rubber productivity in self-rooting JCs. The transcriptome technique allowed transcription changes to be surveyed without prior assumption about which genes might be differentially expressed in the latex between self-rooting JCs and DCs. A total of 1716 genes were found to be differentially expressed, of which 906 showed significant homology to genes with known or partially known functions. The 906 genes belong to different functional categories, indicating that self-rooting JCs and DCs differ in various physiological and biochemical pathways.

The efficiency of sugar transportation and metabolism closely associates with the ability of latex regeneration between interval of successive tappings (Tang et al., 2010; Chao et al., 2015; Liu et al., 2015). The sucrose content in the latex reflects the balance of sucrose loading and utilization in the laticifers. Sucrose is one of the limiting factors for rubber production (Tang et al., 2010; Liu et al., 2015). However sucrose utilization is always limited by the activity of a neutral/alkaline type of invertase (Tang et al., 2010). A previous report indicated that the source content was significantly higher in the latex of self-rooting JCs than in the latex of DCs (Yuan et al., 1998). In the present study, the upregulation of seven unigenes encoding sugar transporters and three unigenes encoding neutral/alkaline invertase suggests an increased efficiency of sucrose transportation and carbohydrate metabolism in self-rooting JCs. Isopentenyl pyrophosphate (IPP) is biosynthesized through the mevalonate (MVA) pathway and runs several enzymatic reactions to form the final high-molecular-weight rubber molecule (Chow et al., 2007). The expressional regulation of the genes in the MVA pathway is directly involved in rubber biosynthesis (Sando et al., 2008). Several rubber-biosynthesis genes, such as 3-hydroxy-3-methylglutaryl coenzyme A reductase (Chye et al., 1992), rubber elongation factor (REF) (Dennis and Light, 1989), small rubber particle protein (Oh et al., 1999), and rubber transferase or *cis*-prenyltransferase (CPT) (Asawatreratanakul et al., 2003), have been extensively studied, and the expression of REF and CPT in the latex was even found to correlate positively with the productivity of rubber tree varieties (Chen et al., 1994; Priya et al., 2007). In this study, the differential expression of rubber-biosynthesis genes in the latex of self-rooting JCs and DCs accounted little for the higher rubber yield of the two clones, suggesting that the higher rubber yield characteristics of self-rooting JCs are slightly correlated with the expression of rubber-biosynthesis genes but are associated with the differential expression of the other genes involved in sugar availability and catabolism, as well as other kinds of metabolisms within the latex cells.

The latex productivity of a rubber tree depends mainly on the duration of latex flow after tapping and the capability of latex regeneration between two consecutive tappings (Tang et al., 2013; Chao et al., 2015). In addition, signaling pathways, especially those of ethylene, jasmonate, and wounding, are actively implicated in the regulation of latex regeneration (Zhu and Zhang, 2009; Duan et al., 2010; Tang et al., 2013). In this study, four unigenes encoding jasmonate signaling components, including lipoxygenase, allene oxide cyclase, jasmonate *O*-methyltransferase, and jasmonate ZIM-domain, as well as two unigenes for the ethylene signaling pathway, were upregulated, and at least 12 genes related to the AP2-EREBP family are more highly expressed in self-rooting JCs than in DCs. Moreover, the upregulation of two unigenes encoding aquaporins was noted in this study. One aquaporin (HbPIP2;1) has been confirmed to play a role in regulating the water conduction between the laticifers and the inner liber tissues, and its expression is correlated positively with the ethylene stimulation of latex yield (Tungngoen et al., 2009), suggesting that the upregulation of aquaporins contributes to an improved latex flow, resulting in the higher rubber productivity in self-rooting JCs. Additionally, the duration of latex flow is reported to be dependent on the integrity of lutoids. The presence of NADPH oxidoreductase, which leads to the formation of ROS, has been reported in lutoids. The release of ROS is claimed to be responsible for the peroxidative degradation of lutoid membrane fragility. The damaging of the lutoid membrane leads latex coagulation processes (Chrestin et al., 1997). After tapping, the bursting of lutoid particles leads to the release of hevein, chitinase, and glucanase. These protein inclusions are effective in rubber particle aggregation (Wang et al., 2013). In the present study, unigenes encoding SOD, thioredoxin, peroxidase, and NADH dehydrogenase were upregulated in self-rooting JCs; three unigenes encoding for chitinase and one unigene for glucanase were also upregulated in self-rooting JCs. Overall, these results suggest that self-rooting JCs demonstrate better capability to latex regeneration and the duration of latex flow, thus displaying a phenotype of higher rubber productivity.

Plant regeneration via *in vitro* culture can induce epigenetic variation (Zhang et al., 2014; Machczyńska et al., 2015). Self-rooting JCs derived from somatic embryos of anthers of *H. brasiliensis* were obtained through primary SE. SE is a highly dynamic developmental process involving active dedifferentiation of somatic cells, followed by the induction and maturation of embryos. The process of induction of *in vitro* embryos is mainly regulated by plant growth regulators and stress responses. In addition, these factors may contribute to induce epigenetic modifications during SE (Machczyńska et al., 2015). Changes in DNA methylation or demethylation were frequently observed during SE of *H. brasiliensis* (Li et al., 2015). Cytosine DNA methylation/demethylation is one of the well-studied epigenetic modifications in higher plants and is involved in the regulation of gene expression during developmental processes (Feng and Jacobsen, 2011). Histone acetylation/deacetylation and methylation/ demethylation are important post-translational histone modification processes that regulate gene expression patterns during plant development, as well as in response to various environmental conditions (De-la-Peña et al., 2015). In present study, several genes encoding epigenetic modification enzymes were differentially expressed between self-rooting JCs and DCs, including DNA (cytosine-5)-methyltransferase, chromomethylase, histone acetyltransferase, histone deacetylase, histone-lysine *N*-methyltransferase, and histone demethylase. These results suggest that epigenetic modifications may lead to gene differential expression between self-rooting JCs and DCs.

## Conclusion

**FIGURE 6:**
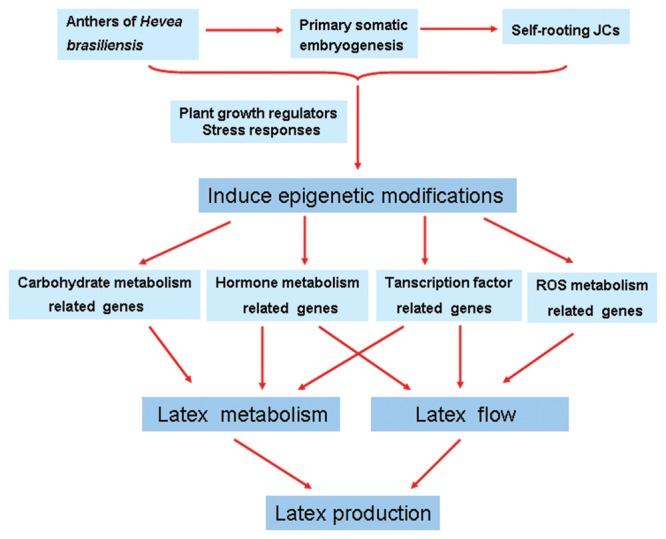
**Schematic of possible mechanisms of increased rubber yield in *Hevea brasiliensis* self-rooting juvenile clones based on the presented data.** Plant growth regulators and stress responses induce epigenetic modifications during SE. Epigenetic modifications may lead to gene differential expression between self-rooting JCs and DCs. Up-regulated expression of carbohydrate metabolism, hormone metabolism, and transcription factor-related genes in the latex, which improved the supply of carbon and energy for latex metabolism. In addition, the up-regulated expression of ROS scavenging, hormone metabolism, and transcription factor-related genes can improve the latex flow.

This study is the first to profile the global gene expression between self-rooting JCs and DCs by using Illumina sequencing technology. Comparative transcriptome analysis revealed new cues at the molecular level for the difference in latex regeneration and duration of latex flow between self-rooting JCs and DCs (**Figure 6**). The profiles of the large number of differential expression genes identified in this work suggest that the self-rooting JCs provide sufficient molecular basis for its higher rubber yielding, especially in the aspects of improved latex metabolisms and latex flow. Epigenetic modifications may lead to gene differential expression between self-rooting JCs and DCs. Nevertheless, the present results provide a valuable guide to understand the molecular mechanism underlying the increased rubber yield of *H. brasiliensis* self-rooting clones.

## Author Contributions

H-LL designed and performed the experiment and drafted the manuscript. S-QP conceived and designed the study and drafted the manuscript. DG, J-HZ, YW, and X-TC prepared samples and analyzed part of the data. All authors read and approved the final manuscript.

## Conflict of Interest Statement

The authors declare that the research was conducted in the absence of any commercial or financial relationships that could be construed as a potential conflict of interest. The reviewer CT declared a shared affiliation, though no other collaboration, with the authors to the handling Editor, who ensured that the process nevertheless met the standards of a fair and objective review.

## Footnotes

1 http://trinityrnaseq.github.io/

2 http://www.ncbi.nlm.nih.gov/

3 http://www.genome.jp/kegg/
